# Curricular Models and Learning Objectives for Undergraduate Minors in Global Health

**DOI:** 10.5334/aogh.2963

**Published:** 2020-08-19

**Authors:** Kathryn H. Jacobsen, M. Cameron Hay, Jill Manske, Caryl E. Waggett

**Affiliations:** 1 George Mason University, Fairfax, VA, US; 2 Miami University, Oxford, OH, US; 3 University of St. Thomas, St. Paul, MN, US; 4 Allegheny College, Meadville, PA, US

## Abstract

**Background:**

A growing number of institutions of higher education offer undergraduate educational programs in global health.

**Objective:**

To identify all undergraduate minors in global health being offered in the United States during the 2019–20 academic year, categorize the curricula being used by secondary programs of study, evaluate the content of required foundational courses, and examine the types of experiential learning opportunities that are offered.

**Methods:**

A working group of the Consortium of Universities for Global Health (CUGH) conducted a systematic review of the websites of all accredited 4-year colleges and universities, identifying 84 institutions offering general global health minors.

**Findings:**

A typical global health minor consists of one introduction to global health course, one epidemiology or health research methods course, several additional required or selective courses, and one applied learning experience. Within this general structure, five curricular models are currently being used for global health minors: (1) intensive minors composed of specialty global health courses, (2) global public health minors built on a core set of public health courses, (3) multidisciplinary minors requiring courses in the sciences and social sciences, (4) anthropology centric minors, and (5) flexible minors.

**Conclusions:**

CUGH recommends ten undergraduate student learning objectives in global health that encompass the history and functions of global health; globalization and health; social determinants of health; environmental health; health and human rights; comparative health systems; global health agencies and organizations; the global burden of disease; global health interventions; and interdisciplinary and interprofessional perspectives.

## Introduction

A diversity of colleges and universities in the United States now offer undergraduate majors and minors in public health, global health, and other areas related to population health [1]. While several recent papers have described undergraduate global health programs at selected sets of institutions [2,3,4,5], and enumerated the graduate global health programs in North America [6,7], no previous papers have attempted to systematically identify and characterize all of the undergraduate global health education programs in the United States. The Consortium of Universities for Global Health (CUGH) pools expertise from 185 member institutions to support global health training, research, practice, and advocacy. One of the goals of CUGH is to support excellence in global health education by developing resources for curriculum design, teaching, and learning. A working group within CUGH that represented diverse institutions and disciplinary backgrounds sought to identify all undergraduate educational programs in global health being offered during the 2019–20 academic year. Minors were the most commonly offered global health programs at the undergraduate level [1], so we categorized the curricula being used by minors, evaluated the content of required foundational courses, and examined the types of applied learning experiences that are required. This paper presents the results of this systematic process and the related recommendations for undergraduate global health learning.

## Methods

We acquired a list of all 4324 colleges and universities that were included in the 2018 Carnegie Classification of Institutions of Higher Education (version 7, released 24 May 2019) produced by the Indiana University Center for Postsecondary Research. Of these schools, 2486 award bachelor’s degrees. We searched the website of each of these 4-year institutions during the 2019–2020 academic year to determine whether the school appeared to offer a major, minor, concentration, field, certificate, or other program in global health or a closely related field. Many schools provide a list of majors and minors in an easily accessible location on their websites. When we were unable to locate such a list, we consulted the school catalog or bulletin and/or the webpages for relevant departments. We minimized our risk of overlooking relevant programs by validating our searches of institutional websites with general search engine queries pertaining to global health majors, minors, concentrations, and certificates.

### Eligibility criteria: minors

We defined a minor as a secondary program that complements a primary area of study in a bachelor’s degree program. We classified secondary programs of study requiring at least 15 semester credit hours (or the equivalent in quarter hours, course counts, or other institutional-specific terms) as minors. Curricular pathways requiring fewer than 15 semester hours or the equivalent, such as short-term study abroad programs and certificates requiring only three courses (equivalent to 12 semester hours or fewer), were not considered to be minors. Global health concentrations embedded within minors in other disciplines (such as public health) or interdisciplinary areas (such as global studies) were included when more than half of the credits in the minor were allocated to the global health concentration. In this paper, we use use the term minor for all programs that met the inclusion criteria, even though some schools call these secondary programs certificates or use other names for them.

### Eligibility criteria: disciplines

Global health is still in the process of being defined as an area of academic study, but it is generally considered to be distinct from public health, which tends to have a focus on health promotion in one community or one nation [8,9]. To be eligible for inclusion in the analysis, a minor had to be focused on global health (rather than public health, global affairs, international development, medical anthropology, population health, or other areas that overlap with global health but do not have global health as the central focus) and cover global health broadly rather than focusing on just one specialty area within global health (such as maternal and child health). Most of the included programs were named global health (44), global public health (13), or global health studies (9). Other program names included community and global health; global health and health policy; global health and social medicine; public health: global health; global and comparative public health; global health and development; global health and humanitarian assistance; global health promotion; global health service; global health technologies; global health, culture, and society; global public health and epidemiology; global public health and the common good; international health; medicine, health, and society: global health; public and global health; and social justice in global health.

### Data sources

For each institution with a global health minor that met our inclusion criteria, we acquired location, institutional enrollment, and other information from the Carnegie Classifications Data 2018 public data file (version 8, released on 6 September 2019) produced by the Indiana University Center for Postsecondary Research. We accessed the curricular and co-curricular requirements for the minor and extracted key details about required courses (title, course level, and hosting department), the thematic areas in which students were required to select one or more courses from a list of approved courses (such as students being required to select one research methods course from a list of two or more preapproved courses), and co-curricular requirements such as field experiences, research or capstone projects, or synthesis capstone courses. Since most of the minors required a course that introduced the fundamentals of global health, we also acquired the course descriptions for each required introduction to global health course from the catalog (or bulletin) or program website of each school.

### Data analysis

We applied grounded theory to the coding of each required element of the curricula of included minors, using several rounds of individual coding, group discussion, and recoding to reach consensus about the codes for each curricular element. The codes noted the disciplinary area for each course (such as biology or statistics), the type of course (such as introductory, methodological, or experiential), and the specific focus (such as environmental health or medical anthropology). Related codes were grouped into categories. We then looked for patterns in the sets of categories that were required curricular components, seeking to identify themes that aligned with three or more institutions. A final set of five curricular models is presented in the results section.

We used a similar process to code the course descriptions of introductory global health courses and establish interrater reliability. To ensure the comprehensiveness of our coding, we separately coded three specific content areas for each course: (1) health and disease topics, such as environmental health, HIV/AIDS, reproductive health, nutrition, noncommunicable diseases, and violence; (2) populations, agencies, and organizations, such as children, low-income countries, refugees, women, health systems, and the World Health Organization; and (3) theories and themes, such as globalization, social justice, and multidisciplinarity. We then reviewed the codes, categorized them, and identified the themes that were most often expressed in the course descriptions.

## Results

Our search identified 84 colleges and universities that offered minors in global health during the 2019–20 academic year, including three schools for which two different programs met our definition of minors in global health (for a total of 87 minors). All 84 schools were nonprofit 4-year institutions, but they represent diversity within this sector of higher education. They were nearly equally divided between 41 public universities and 43 private schools. Of the 84 institutions, 60 were doctoral universities, 12 were master’s universities, and 12 were baccalaureate colleges (all of which were designated in the Carnegie Classifications as awarding more than half of their bachelor’s degrees in arts and science fields rather than awarding the majority of their degrees in professional areas such as business and engineering). Forty-seven of the schools had a large undergraduate enrollment (≥10,000), 19 medium (3000–9999), 17 small (1000–2999), and 1 very small (<1000). In total, 57 of the schools had undergraduate admissions rates that were designated in the Carnegie Classifications as “more selective” (the 80^th^ to 100^th^ percentile of selectivity among undergraduate institutions, based on SAT and ACT percentiles and the admission rate), 22 as “selective” (the 40^th^ to 80^th^ percentile of selectivity), and 5 as “inclusive.” Based on the four location classifications used by the National Center for Education Statistics (NCES), 52 of the schools were in cities, 13 in suburbs (that is, within urbanized areas but outside of principal cities), 19 in towns (outside of urbanized areas), and none in rural areas.

Many of the schools with global health minors offered other health-related educational programs, including 43 offering an MPH degree (including 12 with an MPH concentration in global health), 36 offering an undergraduate major in public health, 33 offering a medical degree (MD, DDS, DMD, DO, or DVM), and 24 offering an undergraduate minor in public health. Thirteen offered an undergraduate major in global health, and at least a dozen additional schools offered a secondary major in global health or a concentration in global health within another major, such as public health or global studies.

### Required coursework

An introduction to global health course was required by 80% (70/87) of the included minors. The interdisciplinary nature of global health was evident in the prefixes for the 69 introductory courses (one of which was required by two different minors at the same institution). In addition to catalog prefixes specific to global health, public health, health science, health administration, and other health fields, these introductory courses were also offered by Africana studies, anthropology, biology, international studies, kinesiology, nursing, nutrition, political science, and sociology departments as well as by interdisciplinary programs.

The topical themes featured most frequently in the descriptions of introduction to global health courses included the social and environmental determinants of health; the global burden of disease, often described using terms such as epidemiology, demography, data, and methods; global health agencies and organizations; governance and comparative health systems; and interventions related to exposures, diseases, and special populations. The conceptual themes that were expressed most often in introductory global health courses (that is, the cross-cutting themes that were not consistently associated with specific exposures, diseases, and populations/groups) included multidisciplinarity or interdisciplinarity; globalization; health disparities and inequalities; ethics, human rights, and social justice; problem solving; and the historical context for global health today.

The introductory course was the only curricular component required by a strong majority of programs. About half of the current global health minors (39/87) required at least one course on epidemiology, statistics, and/or the research methods used in public health or the social sciences. Other popular clusters in which courses were required for global health minors included, in decreasing frequency, courses on health, culture, and society; the biology of health and disease; health policy and economics; and environmental health and sustainability; and ethics, human rights, and social justice.

Thirty-eight (44%) of the 87 global health minors required some type of capstone experience or another type of applied or experiential learning. While some of the 38 programs mandated that all students in the minor complete the same type of capstone experience—such as all students completing a practicum or internship (7), a research project (5), a capstone course with an experiential component, such as service-learning or problem-based learning (4), study abroad (3), an applied project (3), or a reflective portfolio (2)—the most common option was to allow students to choose from a list of approved experiential learning activities (14). Only five minors required an international experience.

**Figure 1:**
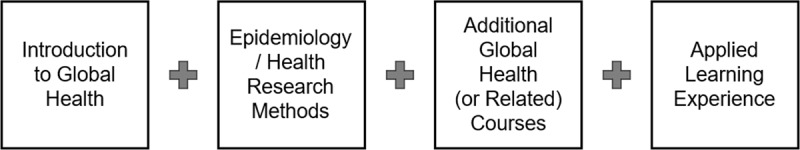
General Structure for a Minor in Global Health.

Based on the most frequently required curricular elements, the general framework for a global health minor includes one introduction to global health course, one epidemiology or health research methods course, several additional courses exploring different domains of global health, and some type of applied learning experience (Figure 1). However, few schools strictly follow that model in its entirety. There is considerable variability in which domains of global health are emphasized and how rigid the curricula are. At some schools, every student who earns a global health minor completes the same set of five (or more) courses; at some other schools, students select their own courses for the minor from a long list of “selective” courses.

### Curricular models

Based on our evaluation of the curricular structures of the global health minors, we identified five curricular models being used for global health minors (Table 1). *Intensive global health* minors are built around a series of specialty courses in global health, such as courses on global environmental health, global health policy and systems, and global health leadership. Most of the 21 universities offering this track were doctoral universities with large undergraduate enrollment. The majority of the intensive global health minors were offered by a school of public health or health professions that also offers an undergraduate major, master’s degree, or graduate certificate in global health. About half (10/21) of the intensive minors required some type of capstone experience, most often a practicum.

**Table 1:** Current Curricular Models for Global Health Minors (2019–20).

Model	Description	Typical Institutions

Intensive	An intensive global health minor typically consists of a series of specialty courses within global health (such as courses on global environmental health, maternal and child health, and global health leadership)	Universities offering an undergraduate and/or graduate degree, concentration, or certificate in global health as well as both bachelor’s and master’s degrees in public health
Global public health	A global public health minor is centered around a core of three or more introductory public health courses, typically including public health, epidemiology, and global health	Colleges and universities that offer public health degrees at the undergraduate and/or graduate level but offer limited opportunities for global health engagement
Multidisciplinary	A multidisciplinary global health minor requires students to select courses from a diversity of academic departments, including at least one course offered by a science department (usually biology or environmental science) and at least one course offered by a social science department (such as sociology)	Liberal arts colleges and universities that do not offer undergraduate or graduate public health degrees
Anthropology	A social science oriented global health minor typically requires several courses related to anthropology, sociology, culture, and related fields	Universities for which the undergraduate minor is the only global health education program
Flexible	A flexible minor typically consists of an introductory global health course plus several electives selected from a generous list of science and/or social science fields	Liberal arts colleges and universities that do not offer undergraduate or graduate public health degrees

*Global public health* minors are built on the three building blocks for undergraduate public health education that were published by the Association of American Colleges and Universities (AAC&U) and the Association for Prevention Teaching and Research (APTR) in 2008: introductory courses in public health, epidemiology, and global health [10]. Global public health minors were offered by 19 schools, including 12 that include “public” in the names of their global health minors. Global public health programs were often housed in departments or schools of public health at universities that did not offer global health majors. Only a few (4/19) global public health minors had an experiential learning requirement.

*Multidisciplinary global health* minors require students to take courses from several departments to fulfill course requirements, including at least one natural science course (typically biology or environmental science) and at least one social science course. Multidisciplinary minors were offered by 14 schools, including six of the 10 baccalaureate colleges and eight of the 12 small schools. Their program descriptions typically emphasized the value of learning about health from a diversity of disciplinary perspectives. Most of these schools did not offer undergraduate public health majors or minors or MPH degrees. Multidisciplinary minors were often hosted by the interdisciplinary or interdivisional unit of a college of liberal arts and sciences rather than being overseen by one academic department. More than half (8/14) of the multidisciplinary minors required some type of a capstone experience, with students typically being allowed to choose from a variety of approved experiential learning opportunities.

*Anthropology global health* minors require several courses in anthropology, sociology, and related disciplines. This type of minor was offered by eight schools, including three with “social justice” or “social medicine” in the names of their global health minors. Most of these programs required at least one course in medical anthropology or a closely related area, and several include an introductory anthropology course among the requirements for the global health minor. Most of these programs were housed in anthropology departments or in social science divisions at public doctoral universities that did not offer any other baccalaureate or graduate programs in global health. Experiential requirements were not common (2/8) in anthropology global health minors.

A *flexible global health* minor built from elective courses is offered by 25 schools. These programs typically require an introductory global health course and some sort of capstone experience related to global health (such as a research project, experiential learning activity, or seminar), but they allow students considerable flexibility in choosing the other courses they take for the minor. Flexible minors are different from multidisciplinary minors because they do not have distribution requirements spanning several mandated areas of study. Most flexible minors were offered at schools that do not offer a public health major or minor. Flexible minors were housed in interdisciplinary units, colleges of liberal arts and sciences, special centers focused on health or global studies, and health professions schools. Most (14/25) of the flexible minors required a specific type of required capstone experience, such as a study abroad, a capstone course, a research project, or a reflective portfolio.

## Discussion

### Undergraduate global health learning objectives

One of the major challenges for global health as an academic discipline is the lack of national or international standards for what content should be included in the curriculum. For schools that offer global public health, multidisciplinary, anthropology, and flexible global health minors, it is critical for the introductory global health course to provide a comprehensive overview of global health theory and practice that equips students with a broad knowledge foundation that they can build on when they take advanced or elective courses within the minor. Our analysis of the content of introductory global health courses found that most courses cover a similar set of concepts and subject matter content. However, there was no standard set of learning objectives available to schools that were creating new global health courses or revising existing ones.

In our role as members of the CUGH working group tasked with developing evidence-based resources for undergraduate education in global health, we decided to use our analysis of current course content and our familiarity with existing resources for teaching and learning in global health as a foundation for a draft list of candidate learning objectives for global health survey courses in North American colleges and universities. Two organizations’ documents provided a starting point for prioritizing content. One is CUGH, which has developed interprofessional global health competencies that span levels from “global citizen” to “advanced.” [11] The lower-level competencies that focus on understanding global health theories and preparing for experiential learning with host organizations that serve people with different cultural and socioeconomic backgrounds are appropriate targets for undergraduates [11]. The other is the Council on Education for Public Health (CEPH), which accredits public health degree programs. CEPH has identified essential components of undergraduate public health education and created a list of foundational knowledge areas in public health [12,13]. While the CEPH accreditation standards do not specifically address global health, they outline areas of undergraduate study that prepare students for entry-level practice and graduate studies in various areas of the population health sciences [14]. We also examined the competencies for global health education proposed by international groups for students and trainees in medicine, public health, and other areas [15]. Together, the educational frameworks of CUGH, CEPH, and international groups point toward potential priorities for undergraduate global health courses and programs.

Based on the categories and themes we identified in our analysis of the current content of introductory courses required by existing global health minors in 2019–20, our review of existing educational frameworks related to global health [11,12,15], and expert feedback from CUGH members, we generated a preliminary list of recommended student learning objectives for an introductory global health course. This list included central global health principles, such as globalization and health equity, plus foundational knowledge areas related to the determinants of health, the major causes of morbidity and mortality, and the entities involved in funding and implementing global health interventions. After several rounds of revision and refinement, a final set of ten CUGH Recommended Undergraduate Global Health Student Learning Objectives was reviewed by members the CUGH Education Committee and the CUGH Secretariat, and then endorsed by CUGH (Table 2).

**Table 2:** Recommended Undergraduate Global Health Student Learning Objectives.

#	Learning Objective

1	Describe the history, values, and functions of global health.
2	Explain how travel, trade, and other aspects of globalization contribute to health, disease, and health disparities.
3	Summarize the social, economic, cultural, and political contributors to individual and population health.
4	Examine the connections between human health and environmental health, including considerations of water, sanitation, air quality, urbanization, and ecosystem health.
5	Discuss the relationship between human rights and global health.
6	Compare the financing and delivery of medical care in countries with different types of health systems and different income levels.
7	Evaluate the roles, responsibilities, and relationships of the agencies and organizations involved in financing and implementing public health interventions locally and internationally.
8	Compare the burden of disease, disability, and death from infectious diseases, nutritional deficiencies, maternal and perinatal conditions, noncommunicable diseases, mental health disorders, and injuries in countries with different income levels.
9	Identify evidence-based, cost-effective, sustainable interventions for promoting health and preventing illness across the lifespan from the prenatal period through older adulthood.
10	Apply an interdisciplinary or interprofessional lens to the evaluation of policies and interventions that seek to solve major population health concerns and achieve health equity.

The CUGH Recommended Undergraduate Global Health Student Learning Objectives are not meant to be an exhaustive list of all global health principles and themes. However, including these ten items alongside institution-specific learning goals and competencies will ensure coverage of critical global health knowledge and skill areas. These learning objectives are applicable to introductory global health courses housed in diverse academic units. If the verbs used for each item (such as define, explain, compare, and evaluate) are scaled up to higher order thinking skills, these items could also form the basis for program-level learning goals for an entire global health program, such as a certificate, minor, or major.

Rather than dictating the specific topics that a survey course should address—like naming the exact diseases and populations that should be examined in an introductory course—these learning objectives provide a framework for exploring a variety of global health issues. For example, although pandemics are not directly named within these ten items, the components of the emergency management cycle (prevention, preparedness, response, and recovery) align with many of the learning objectives. Global health security is one of the functions of global health (learning objective #1). Pandemics arise from globalization (#2) as well as from human behavior (#3) and environmental interactions (#4). The responses to pandemics, such as mobility restrictions, may raise questions about human rights (#5). Pandemics like COVID-19 place a heavy burden on medical care providers (#6) and require substantial investment in public health interventions like contact tracing (#7). Case studies about the coronavirus pandemic provide opportunities to compare the epidemiological profiles of different populations (#8), examine which interventions are effective in various demographic groups (#9), and think creatively about what policies and practices might prevent another emerging infectious disease pandemic (#10). While students will benefit from exposure to a wide range of health issues, not just one, in their introductory global health courses, these learning objectives allow the flexibility to adapt courses to emerging health issues and local priorities.

### Curricular models for global health minors

In many academic disciplines, a minor comprises a subset of courses from the corresponding major. For example, the American Psychological Association provides strong guidance about the knowledge and skill components of foundational, intermediate, advanced, and capstone courses in a psychology major, and it recommends that a minor require completion of about four foundational courses rather than being based on one introductory psychology course plus higher-level coursework [16]. The American Chemical Society’s educational standards list the specific subdisciplines in which coursework is required for a major in the field [17], and although ACS has issued no standards for minors in chemistry, it is typical for minors to consist of the foundational courses plus a subset of the advanced courses from the major. However, this “minors as mini-majors” approach will not work in global health, since few schools currently offer global health majors and there is not yet consensus about what types of courses are important for a major in global health [1].

When minors are not derived from majors, they are often designed around interdisciplinary themes. For example, public health minors offered by diverse types of institutions often consist of three foundational courses (epidemiology, U.S. public health, and global health) plus a few “selective” courses (that is, courses selected from lists of approved course options) and at least one experiential learning activity [18,19]. This structure is similar to the curricular plan used by many global health minors (and featured in Figure 1). We identified five variants of this general structure that are currently being used for global health minors. These models are characterized based on whether they are built around specialty courses in global health (intensive) or public health (global public health), require taking courses in several different departments (multidisciplinary) or primarily in one department (anthropology), or are relatively unstructured (flexible).

Each of the five curricular models used by global health minors has its own set of curricular and programmatic strengths and weaknesses (Table 3). Minors that align with the intensive and global public health models may provide the most comprehensive exposure to global health principles and practices, but they require substantial institutional investments in courses specific to the minor. These resource demands make these models most suitable for large schools that already offer many courses on global health topics as part of a public health or global health major. Minors that align with the multidisciplinary or flexible models may allow students the greatest freedom to individualize their studies to match personal interests and goals, but even with strong advising these loosely-structured programs may leave students with critical gaps in their understanding of global health.

**Table 3:** Evaluation of Current Curricular Models for Global Health Minors (2019–20).

Model	Strengths	Possible Limitations

Intensive	Allows for deep exploration of specific areas within global health (such as global environmental health, global health policy, emerging infectious diseases, maternal and child health, and global health leadership)	Requires significant institutional resources specific to global health; Courses taught by experts in focused areas of research and practice may offer depth at the expense of disciplinary or professional breadth
Global public health	Emphasizes a set of core public health courses (introductory courses in epidemiology, global health, and U.S. public health policy) for which resources are available to support instructors without specialized training in global health	Treats global health as a subdiscipline of public health rather than as a multidisciplinary field that overlaps public health while drawing on other academic and professional areas; The public health core leaves few credits for courses specific to global health
Multidisciplinary	Applies a liberal arts lens to global health education by requiring students to examine complex issues from the perspectives of the natural sciences, social sciences, and (sometimes) the humanities; Requires limited institutional investment in courses specific to the global health minor	Demands that a single introductory global health course cover a wide range of global health principles and practices, because electives in supporting disciplines might only peripherally touch on topics specific to global health (such as a course in environmental science only briefly examining global environmental health concerns)
Anthropology	Allows students majoring in fields other than anthropology to deeply understand anthropological theories and methods as they apply to global health	Focuses narrowly on anthropological (and sometimes also sociological) approaches to global health, providing limited exposure to biological and public health perspectives
Flexible	Allows students the greatest freedom to craft their own programs of study that best align with their academic and professional interests; Requires limited institutional investment in courses specific to the global health minor	Requires intensive advising to ensure that each student’s unique set of curricular and co-curricular learning experiences provide some sort of coherent engagement with global health and enable the student to synthesize, integrate, and apply central global health principles

### Recommendations for global health minors

Given the diversity in course requirements across the current minors, even when looking at minors that apply the same general type of curricular model, it would be premature to recommend a set of standard courses for global health minors. However, all courses that count toward a minor should build on the undergraduate student learning objectives introduced in the fundamentals course, and all should have health as a central theme. Schools lacking the resources to offer a variety of specialized global health courses should still verify that all courses that count toward the minor engage with global health in a meaningful way. General courses in anthropology, biology, communication, economics, environmental science, government and policy, philosophy, sociology, and other areas are unlikely to provide sufficient engagement with global health themes; by contrast, health-specific courses such as medical anthropology, health communication, and environmental health are likely to engage more closely with global health principles and practices.

Similarly, applied learning experiences completed as part of global health minors should require students to synthesize and reflect on the global health knowledge areas and competencies spelled out in the undergraduate student global health learning objectives. Many types of experiential learning opportunities may be suitable for undergraduates [20]. Experiential learning does not require international travel, and it can involve student engagement with a diversity of clinical and non-clinical professional practice areas within the governmental, nonprofit, and commercial sectors. (Mentors should ensure that students do not perform beyond the scope of their training, especially in clinical settings.) Schools where institutional resources are not available to offset the burden that experiential learning activities can place on students, faculty mentors, and community hosts may opt not to require applied learning experiences for all students minoring in global health, but they can still recommend experiential learning as an optional curricular component.

### Study limitations

Our results imply that 3.4% (84/2486) of all 4-year colleges and universities offered minors in global health in 2019–20, but since some schools that award bachelor’s degrees do not offer minors in any discipline, the true percentage is greater than 3.4%. We used a systematic approach to attempt to identify all the colleges and universities offering global minors in 2019–20, but it is likely that our months-long review process missed some programs. Global health is a recently launched curricular offering at many schools, and some websites might not have been updated at the time we conducted our review of their institutional webpages. The curricular trends we observed across 87 general global health minors are unlikely to be affected by one or more programs being overlooked.

Our count of minors in global health intentionally omits minors in specialty fields related to global health and minors in areas such as public health, population health, and medical anthropology that overlap with global health but are distinct fields of study. If our eligibility criteria had used a broader definition of global health, there would be well over 100 minors included in the tally. The count also intentionally excludes several new global health minors that were announced in 2020 or earlier but were not beginning enrollment until the 2020–21 academic year.

## Conclusion

This analysis provides a snapshot in time of the institutions of higher education in the Untied States that were offering global health minors during the 2019–20 academic year along with the content they included in their introductory courses, the curricular models they were using for the remaining courses in the minor, and the types of applied learning experiences they required. It also introduces the CUGH Recommended Undergraduate Global Health Student Learning Objectives, summarizes the general framework used by the typical global health minor, and describes the models that schools use to structure their minor curricula. We expect that the number of schools offering global health minors will increase over the next few years in response to student demand driven by the coronavirus pandemic as well as by other ongoing global challenges, such as climate change. The recommended learning objectives and curricular framework presented in this paper provide schools with tools they can use to design minors that align with disciplinary norms and with institutional goals, values, and resources.
